# Redirecting Metabolic Flux towards the Mevalonate Pathway for Enhanced *β*-Carotene Production in *M. circinelloides* CBS 277.49

**DOI:** 10.1155/2020/8890269

**Published:** 2020-12-29

**Authors:** Tahira Naz, Yusuf Nazir, Shaista Nosheen, Samee Ullah, Hafiy Halim, Abu Bakr Ahmad Fazili, Shaoqi Li, Kiren Mustafa, Hassan Mohamed, Wu Yang, Yuanda Song

**Affiliations:** ^1^Colin Ratledge Center for Microbial Lipids, School of Agricultural Engineering and Food Science, Shandong University of Technology, Zibo 255000, China; ^2^Department of Food Sciences, Faculty of Science and Technology, Universiti Kebangsaan Malaysia, 43600 UKM Bangi, Selangor, Malaysia; ^3^University Institute of Diet and Nutritional Sciences, Faculty of Allied Health Sciences, The University of Lahore, Lahore 54000, Pakistan; ^4^Department of Botany and Microbiology, Faculty of Science, Al-Azhar University, Assiut 71524, Egypt

## Abstract

Carotenoids produced by microbial sources are of industrial and medicinal importance due to their antioxidant and anticancer properties. In the current study, optimization of *β*-carotene production in *M. circinelloides* strain 277.49 was achieved using response surface methodology (RSM). Cerulenin and ketoconazole were used to inhibit fatty acids and the sterol biosynthesis pathway, respectively, in order to enhance *β*-carotene production by diverting metabolic pool towards the mevalonate pathway. All three variables used in screening experiments were found to be significant for the production of *β*-carotene. The synergistic effect of the C/N ratio, cerulenin, and ketoconazole was further evaluated and optimized for superior *β*-carotene production using central composite design of RSM. Our results found that the synergistic combination of C/N ratios, cerulenin, and ketoconazole at different concentrations affected the *β*-carotene productions significantly. The optimal production medium (std. order 11) composed of C/N 25, 10 *μ*g/mL cerulenin, and 150 mg/L ketoconazole, producing maximum *β*-carotene of 4.26 mg/L (0.43 mg/g) which was 157% greater in comparison to unoptimized medium (1.68 mg/L, 0.17 mg/g). So, it was concluded that metabolic flux had been successfully redirected towards the mevalonate pathway for enhanced *β*-carotene production in CBS 277.49.

## 1. Introduction

Carotenoids are well known bioactive compounds and high-value nutritional molecules that play a defensive role against chronic diseases due to their antioxidant potential [1]. In the modern industry, carotenoids are mainly extracted from plants [2], chemically produced [3], and produced by microorganisms, such as *Phaffia rhodozyma*, *Dunaliella salina*, and *Blakeslea trispora* [4]. The chemical production method is mainly used for commercial production due to low production costs and a well-established synthetic technology route. However, this method is embedded with some drawbacks such as by-products of undesired side effects, and waste generated during the production process is harmful to the environment [5]. Carotenoid production from microbial sources is highly fruitful as microorganisms can easily be accommodated for multiple fermentation processes [6]. However, commercial production of carotenoids using the microbial system could have major limitations such as slow growth, less yield, and high cost involved in the production as compared to chemical production. Nevertheless, this limitation could be overcome by using strain improvement strategies (genetic modification) to optimize growth and carotenoid production.

*β*-Carotene is an orange-yellow lipophilic carotene and known as provitamin A. It has high market value due to its diverse biological functions and extensive uses in pharmaceutical, cosmetics, food, and textile industries [7]. Human intervention studies showed that *β*-carotene is the key factor in the prevention of pharynx oral and larynx cancer, which was confirmed by a high intake of food rich in *β-*carotene resulting in the reduction of risk for throat and oral cancers by about 50% [8].

Commonly three zygomycetes, *Blakeslea trispora*, *Phycomyces blakesleeanus*, and *Mucor circinelloides*, have been reported for carotene biosynthesis of which *β*-carotene is the principal carotenoid [9]. The main benefit of using filamentous fungus over bacteria or yeast is the high levels of metabolite and final biomass production. *B. trispora* and *P. blakesleeanus* are well known for the highest production of carotenoid. However, a few drawbacks are linked with the industrial utilization of these two fungi. Shaking cultivation of *B. trispora* was associated with declined accumulation of carotenoid, so surface cultivation must be performed, and the optimum growth temperature for these zygomycetes should never be above 25°C [10]. Hence, it has been proposed that the industrial production of carotenoid by *M. circinelloides* could be simpler as compared to the complicated fermentation process used for the production of *β*-carotene in *B. trispora* [11]*. M. circinelloides* is considered a true “microbial factory” due to its intrinsic potential to produce enzymes, organic acids, essential amino acids, food pigments, ethanol, chitosan, polyphenols, and microbial lipids [12, 13]. *β*-Carotene production in *M. circinelloides* occurs naturally, and the carotenoid biosynthesis in this fungus is well studied owing to the accessibility of genetic tools that can help in the development of hyper *β*-carotene-producing strain. Furthermore, the dimorphic nature of *M. circinelloides* aids in biotechnological production [14]. Being a model strain of carotenogenic studies, many regulatory investigations have been conducted using *M. circinelloides* [11].

Cost reduction is one of the crucial factors in the fermentation scale-up process for any bioactive compound production. Hence, industrial production of carotenoid would only be feasible if production cost could be minimized by using suitable carbon sources and increasing the product yield [15]. The development of a suitable cultivation medium to obtain a large amount of the desired product with a low-priced substrate is an essential feature of the fermentation process [1]. Medium components could drastically affect the accumulation of *β*-carotene in terms of both overall productivity and relative yield of *β*-carotene in association with cell growth [16]. Apart from substrate optimization, many factors have been investigated for *β*-carotene production, such as environmental conditions [17], the use of stimulator [18], application of oxidative stress [5, 19], and overexpression of carotenogenic genes [18, 20] in various microorganisms.

**Figure 1:**
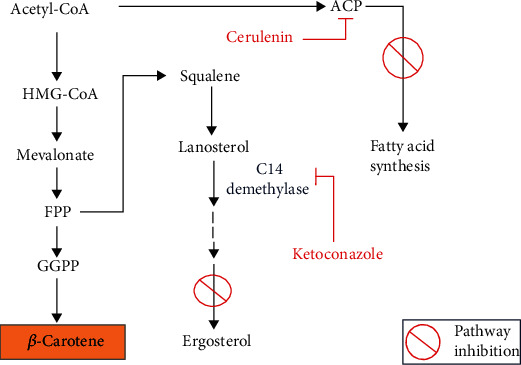
Schematic presentation of carotenoid, sterol, and fatty acid synthesis pathway in *M. circinelloides.*

Our previous finding indicated *M. circinelloides* strain CBS 277.49 as a dominant producer of *β*-carotene than *M. circinelloides* strain WJ11 [21]. So, in the current investigation, strain CBS 277.49 was nominated as a suitable candidate. This study was aimed at optimizing the production of *β*-carotene by screening different carbon sources and chemical modulators to divert metabolic flux of acetyl-CoA and FPP from fatty acid synthesis (FAS) and sterol synthesis pathways towards the mevalonate pathway. Based on a well-established fact that acetyl-CoA is a shared precursor for terpenoid and fatty acid biosynthesis [22], it was proposed that decreased fatty acid production might result in the transfer of more acetyl-CoA to the carotenoid biosynthesis pathway in CBS 277.49. Since both sterols and carotenoid biosynthesis pathway share intermediate farnesyl diphosphate (FPP), redirecting FPP towards the mevalonate pathway could increase the biosynthesis of *β*-carotene by using an ergosterol inhibitor (Figure 1). To confirm our hypothesis, cerulenin was used to block the FAS pathway and ketoconazole was used to inhibit ergosterol biosynthesis in *M. circinelloides* for enhanced *β*-carotene production. There are two different aspects that we considered to achieve substantial *β*-carotene production by CBS 277.49: (1) optimization of medium components by screening different carbon sources and C/N ratios and (2) redirection of metabolic flux towards the mevalonate pathway by using inhibitors of the FAS and sterol pathway, individually or in combination. Many studies have been conducted for the enrichment of *β*-carotene in different microorganisms. To date, an optimization study of *β*-carotene biosynthesis in *Mucor* using statistical analysis has not been studied extensively and required further comprehensive exploration. So, to the best of our knowledge, this is the first study to optimize the production of *β*-carotene in CBS 277.49 by diverting metabolic flux towards the mevalonate pathway from the FAS and ergosterol synthesis pathway using inhibitors of the mentioned pathways.

## 2. Materials and Methods

### 2.1. Fungal Strain and Fermentation Condition

*M. circinelloides* strain CBS 277.49 was used in the present study [23]. Seed culture was prepared by inoculating 100 *μ*L spore suspension (nearly 10^7^ spores/mL) into 150 mL of Kendrick and Ratledge (K&R) medium. This medium contained 30 g glucose, 7 g KH_2_PO_4_, 3.3 g diammonium tartrate, 2 g Na_2_HPO_4_, 1.5 g yeast extracts, 1.5 g MgSO_4_·H_2_O, 0.1 g of CaCl_2_·2H_2_O, 8 mg FeC1_3_·6H_2_O, 1 mg ZnSO_4_·7H_2_O, 0.1 mg of MnSO_4_·5H20, 0.1 mg CuSO_4_·5H_2_O, and 0.1 mg Co(NO_3_)_2_·6H_2_O in one liter of distilled water and was stored in 500 mL baffled flasks to support aeration and incubated for 24 h in a shaking incubator at 28°C and 150 rpm. Then, 10% (*v*/*v*) of this overnight grown culture was inoculated in 150 mL modified K&R media, which contained 80 g of glucose and 2 g/L diammonium tartrate held in a 1000 mL baffled flask for subsequent fermentation under the continuous light. All experiments were repeated three times.

In the first part of this study, different screening experiments were performed to achieve optimized medium concentration to produce *β*-carotene by CBS 277.49.

### 2.2. Screening of Carbon Sources

The fungal strain was cultivated on five carbon sources for enriched *β*-carotene production and growth of CBS 277.49. The carbon sources used for this study were fructose, glucose, glycerol, maltose, and molasses at a concentration of 80 g/L with other medium components. The culture of CBS 277.49 was grown for 72 h under continuous light at 28°C and 150 rpm. After 72 h, fungal biomass was harvested and analyzed for cell dry weight (CDW), total lipid %, and *β*-carotene concentration for all carbon sources.

### 2.3. Screening for Optimal C/N Ratios

The optimal C/N ratio was determined in 1000 mL baffled flask holding 150 ml K&R media. Inorganic salt ammonium tartrate dibasic served as a nitrogen source in our study. Carbon sources were kept constant at 80 g/L while the nitrogen source was adjusted to make C/N ratios of 5, 10, 20, 40, and 80 (*w*/*w*). The effect of different C/N ratios on microbial cell growth, lipid %, and *β*-carotene production was investigated. The control flask did not contain any inorganic nitrogen source.

### 2.4. Effect of the FAS Inhibitor on *β*-Carotene Production

Since the initial addition of cerulenin can significantly affect fungal cell growth, it was introduced into media after cells attained the late exponential phase, i.e., after 12 h of cultivation. Cerulenin was dissolved in ethanol and prepared in the following concentration set, i.e., 5, 10, 20, 40, 80, and 100 *μ*g/mL. The control flask did not contain cerulenin. Fungal cultures were grown in continuous light for 72 h at 28°C. Then, biomass was harvested and investigated for CDW, lipid %, and *β*-carotene.

### 2.5. Effect of the Ergosterol Inhibitor on *β*-Carotene Production

Ketoconazole limits the biosynthesis of ergosterol and increases the accumulation of *β*-carotene by driving more FPP to the carotenoid biosynthesis pathway. Firstly, it was dissolved in ethanol and then added into the fermentation media after 12 h of culture growth. In our study, different concentrations of ketoconazole were added in the following concentrations: 10, 25, 50, 100, 150, and 200 mg/L to find out the optimal level that can inhibit ergosterol biosynthesis and enhance *β*-carotene production by CBS 277.49. The fungal cultures were incubated for 72 h at 28°C under continuous light, and then, biomass was harvested and CDW, ergosterol, and *β*-carotene concentration were determined.

### 2.6. Optimization of Significant Factors by RSM

Production of *β*-carotene in *M. circinelloides* CBS 277.49 was further optimized using Central Composite design of RSM (CCD-RSM) and Design Expert Software (DOE; version 11.0, Stat-Ease, USA) in a 1000 mL baffled flask holding 150 mL K&R media with the variable of factors as presented in Table 1. Three factors, CN ratios, cerulenin, and ketoconazole, were selected to be optimized with the following extent of concentrations (C/N ratios, 10-40 *w*/*w*; cerulenin, 20-50 *μ*g/mL; and ketoconazole, 10-200 mg/L) and cultivated for 72 h (Table 1).

Statistical investigation using ANOVA with the DOE software was conducted to estimate the optimal factors for the maximum production of *β-*carotene by CBS 277.49. Multiple regression analysis of the experimentally attained results was used to calculate the coefficients in the second-order polynomial:

(1) $$\begin{array}{c} Y=b\prime_{0}+\overset{n}{\underset{i=1}{\sum }}b_{i}X_{\text{i}}+\overset{n}{\underset{i=1}{\sum }}b_{ii}X_{i}^{2}+\overset{n}{\underset{i=1}{\sum }}.\overset{n}{\underset{j\geq 1}{\sum }}b_{ij}X_{i}X_{j}, \end{array}$$

where *Y* is the predicted response, *b*_0_′ is the constant coefficient, *b*_*i*_ is the linear coefficient, *b*_*ij*_ is the interaction coefficient, *b*_*ii*_ is the quadratic coefficient, and *X*_*i*_ and *X*_*j*_ are coded values. Besides, three-dimensional (3D) response surface and contour plots were created to visualize the trend of the maximum responses and interaction outcome of significant variables on the responses.

### 2.7. Analytical Methods

#### 2.7.1. CDW Determination

Biomass was collected on a dried and preweighed filter paper by filtration through a Buchner funnel using reduced pressure and washed thrice with distilled water. Then, after freezing at -80°C, the biomass was freeze-dried. The weight of the biomass was gravimetrically calculated.

#### 2.7.2. Lipid Extraction

Total lipids were extracted as defined by Folch et al., with slight modifications [24]. Briefly, 50 mg of lyophilized mycelia was used for total lipid extraction. The weight of the blank lipid extraction tube was measured. Extraction of total lipid was done with chloroform/methanol (2 : 1, *v*/*v*), and after drying using nitrogen, the weight of the tube with lipid was measured. Total lipid % in CBS 277.49 was calculated using the following equation:

(2) $$\begin{array}{c} \left(\frac{\text{Total lipid}\%=\text{T}1-\text{T}0\;}{\text{Wm}}\right)\times 100, \end{array}$$

where T1 is the weight of the tube with lipid, T0 is the weight of the blank tube, and Wm is the weight of mycelia [25].

#### 2.7.3. Extraction and Quantification of *β*-Carotene

Extraction of *β-*carotene was done according to our previous investigation [21]. Briefly, 100 mg of mycelia powder, which was grinded under liquid nitrogen after freeze-drying, was dissolved in 900 *μ*L of hexane, followed by a vortexing step. This extraction step was repeated until the mycelia powder became colorless. Extracts were then collected in a 50 mL falcon tube, mixed, and partitioned in an equal volume of 10% diethyl ether. 2 mL of distilled water was introduced to help the separation of two layers followed by centrifugation at 3200 rpm and 4°C for 8 min. The fraction of petroleum ether was dried under nitrogen.

High-performance liquid chromatography (HPLC) was conducted for *β*-carotene for samples and standard purchased from Sigma. Dried extracts were resuspended in 800 *μ*L tetrahydrofuran added with butylated hydroxytoluene (100 *μ*g/mL). The samples were then subjected to analysis by HPLC immediately. 10 *μ*L of samples were analyzed on the infinity Lab Pro shell C18 column (4.6 × 150, ODS 4 *μ*m). 96% of methanol and 100% methyl-terc-butyl ether were used as mobile phases listed as A and B, respectively. The following gradient of these two solvents was used for the analysis of samples: min/solvent A_%_/solvent B_%_ was 0/99/1, 8/60/40, 13/46/54, 15/0/100, 18/0/100, 21/99/1, and 25/99/1). The flow rate was set as 1 mL/min. The temperature of the column thermostat was adjusted as 35°C, and the diode array detector was used with a detection wavelength of 450 nm (Agilent Technologies, Santa Clara, USA).

#### 2.7.4. Extraction and Quantification of Ergosterol

Ergosterol was extracted according to the previous investigation [26]. In short, 100 mg of mycelia was saponified by the addition of 4 g KOH and 16 mL HPLC-grade methanol for 2 h at 80°C. When the mixture was cooled down, then 10 mL of petroleum ether was used to extract ergosterol followed by evaporation of ether fraction using nitrogen gas. Then, dried sterol was suspended in 700 *μ*L of methanol and analyzed using methanol : water (97 : 3, *v*/*v*) as a mobile phase flowing at a rate of 1 ml/min under isocratic conditions by HPLC (C-18 column; Agilent Technologies, Santa Clara, USA). Ergosterol was visualized using a diode array detector at 280 nm channel. The standard of ergosterol was bought from Sigma (Saint Louis, MI, USA).

#### 2.7.5. Statistical Analysis

All the experiments were carried out in triplicate to get the average value of the data and error bars by ± standard deviation. All statistical analyses were performed using a two-way ANOVA with multiple comparison tests. These calculations were done by using the GraphPad Prism software for Windows (San Diego, CA, USA). *p* < 0.05 was considered statistically significant.

## 3. Results and Discussion

### 3.1. Effect of Carbon Sources

**Figure 2:**
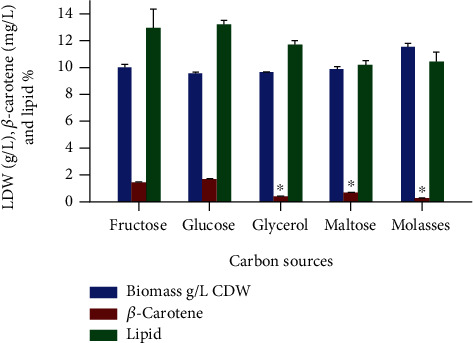
Effect of different carbon sources on the growth, *β*-carotene, and lipid production of *M. circinelloides* CBS 277.49. Values are the mean ± standard deviation of three experiments. Asterisks indicate that the difference is significant in comparison to control (glucose) (^∗^*p* < 0.05).

The selection of suitable carbon sources could have a considerable effect on the overall productivity, peak titer of secondary metabolites, and cost of raw material [27]. During microbial fermentation, a carbon source acts as a significant component for the construction of cellular material and an energy provider as well for microbial growth [28]. All the carbon sources analyzed in our study supported the growth, lipid, and *β*-carotene production to a different extent. As shown in Figure 2, among all the carbon sources assessed, glucose showed maximum production of *β*-carotene 1.68 mg/L (0.17 mg/g) and biomass (9.70 g/L). Fructose was found as a good source for the growth of CBS 277.49, but the production of *β*-carotene was lower than that in glucose, almost 0.8-fold less than that in glucose, i.e., 1.42 mg/L (0.14 mg/g), so glucose was chosen as a carbon source for further experiments. It was also observed that *M. circinelloides* grew well while using maltose as a carbon source but produced a lesser amount of *β*-carotene. However, glycerol and molasses were not found as good carbon sources for *β*-carotene production by strain CBS 277.49 (Figure 2). In few studies, molasses has been reported as the best carbon source for *β*-carotene production [1, 29]. Our result for molasses contrasted with Allam Nafady et al. who found it as the best carbon source for *β*-carotene production followed by glucose in *Fusarium camptoceras* [1]. However, several other literatures reported glucose as the best carbon source for the production of *β*-carotene [16, 30]. Glucose was also documented as the best carbon source in optimizing the production of *β-*carotene by *B. trispora* [31]. Investigation of Jinendiran et al. also discovered that glucose gave a maximum production of *β*-carotene as compared to other carbon sources by *Exiguobacterium acetylicum* S01 [32].

### 3.2. Effect of C/N Ratios

**Figure 3:**
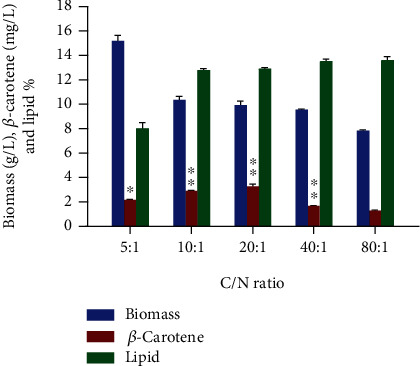
Effect of different C/N ratios on the growth, *β*-carotene, and lipid production of *M. circinelloides* CBS 277.49. Values are the mean ± standard deviation of three experiments. Asterisks indicate that the difference is significant (^∗^*p* < 0.05; ^∗∗^*p* < 0.0001).

Another most critical parameter that influences the development of microbial lipids and carotenoid is the mass balance of carbon and nitrogen sources, in particular cultivation medium improvement [33]. The carbon source and C/N ratio are associated with metabolite and biomass production and affect the secondary metabolite yield [34]. Therefore, various strategies are often employed to find the optimal C/N ratio for enhanced bioactive compounds. The effect of different concentrations of ammonium tartrate dibasic (C/N ratios of 5, 10, 20, 40, and 80) was explored for the production of *β*-carotene. As shown in Figure 3, a lower C/N ratio was found to be more favorable for biomass production by CBS 277.49 (15.2 g/L) but was not found suitable for the accumulation of *β*-carotene. It was observed that the increasing C/N ratio had a definite influence on CDW and the production of *β*-carotene. This finding was following the observation of Elfeky et al. who found the increasing C/N ratio as the most effective parameter for enhanced carotene production [35]. The maximum production of *β*-carotene 3.29 mg/L (0.31 mg/g) was found at a C/N ratio of 20 with biomass of 9.93 g/L. The increasing C/N ratio resulted in the lower nitrogen content in the media. According to Ratledge, limitation of the nitrogen in the media increased the cascade of reactions leading to the formation of acetyl-CoA which then led to a more active fatty acid and terpenoid pathway [36]. This resulted in enhanced *β*-carotene production as shown in Figure 3. This observation is also reported in other study as well [17].

However, with further increase in the C/N ratio to 40 and 80, decrease in cellular *β*-carotene was observed, suggesting that both low and high nitrogen source concentrations could have a negative effect on carotenoid production [33]. Ferrao found the C/N ratio of 10 as an optimal ratio while investigating the effect of different medium constituents on *β*-carotene production in *Rhodotorula graminis* RC04 [16]. Braunwald et al. found a high C/N ratio to be more effective for carotenoid accumulation (1.24 mg/L) in *R. glutinis* [33]. Similar to our results, Gao et al. also recorded a medium to high C/N ratio as the best parameter for improved *β*-carotene in *Yarrowia lipolytica* [18]. More recently, Elfeky et al. reported that carotenoid accumulation inside cells was significantly increased at a high C/N ratio than that of low C/N ratio, regardless of the nature of nitrogen sources used [35].

### 3.3. Effect of Cerulenin on Growth, Total Lipid, and *β*-Carotene Synthesis

Cerulenin is known as a potent antifungal compound that can block fatty acid synthesis in many fungal and yeast strains [37]. Cerulenin interrupts fungal growth by impeding fatty acid synthesis, apparently by inhibiting the fatty acid synthase [38]. It binds irreversibly to fatty acid synthase, specifically b-ketoacyl-acyl carrier protein synthase. It was previously used as a useful compound to control fatty acid composition in *Mycoplasma* species and to investigate the effect of cerulenin on growth [39].

**Figure 4:**
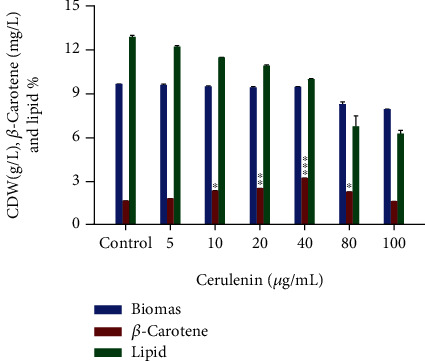
Effect of different concentrations of cerulenin on the growth, *β*-carotene, and lipid production of CBS 277.49. Values are the mean ± standard error of three experiments. Asterisks indicate that the difference is significant in comparison to control (0) (^∗^*p* < 0.001; ^∗∗^*p* < 0.0005; ^∗∗∗^*p* < 0.0001).

In the current study, cerulenin was added in different concentrations such as 5, 10, 20, 40, 80, and 100 *μ*g/mL to achieve enhanced *β*-carotene production without having an adverse effect on cell growth. It was used in very little concentration as it is a potent antifungal drug and can affect fungal cell growth beyond certain levels. The amount of *β*-carotene produced by CBS 277.49 varied widely, depending upon the concentration of cerulenin added, as shown in Figure 4. The growth was not affected significantly but up to a specific limit of cerulenin concentration, after which growth was slightly inhibited. Effectiveness of cerulenin in the enhancement of *β*-carotene accumulation was confirmed by the decline in total lipid production in the cell. As the cerulenin concentration increased, a gradually decreasing trend in lipid production was observed, which corresponded to increased concentration of *β*-carotene, as presented in Figure 4. A significant increment in *β*-carotene 3.23 mg/L (0.31 mg/g) was obtained at a concentration of 40 *μ*g/mL, with a decline in total lipid production up to 10% from 14% by CBS 277.49. Further increasing the concentration of cerulenin was found to inhibit cell growth, and even a decline in *β*-carotene accumulation was also observed, which might be attributed to the cytotoxic effect of the high dose of cerulenin and partial inhibition of HMG-CoA synthase activity [40]. This decline in *β*-carotene accumulation at high cerulenin dose (80 and 100 *μ*g/mL) could also be explained as feedback inhibition at the metabolic level; i.e., high concentration of cerulenin might cause a decline in lipid production (up to 6.3%) in CBS 277.49, resulting in excess of free carotenoid which might inhibit the carotenogenic enzyme, causing a decrease in the de novo synthesis of *β*-carotene [40]. Our screening experiment found a 92% increment in *β*-carotene production by an optimal concentration of cerulenin (40 *μ*g/mL) as compared to the control flask (1.68 mg/L) in which no cerulenin was added. These results concluded that the acetyl-CoA pool was successfully redirected towards the mevalonate pathway from the FAS pathway in CBS 277.49 by using cerulenin.

Miao et al. reported the use of another fatty acid inhibitor, triclosan, and found a two-fold increment in astaxanthin production by a mutant strain of *Phaffia rhodozyma*, termed as MK19 [20]. Ito et al. studied the effect of cerulenin on morphogenesis of *Mucor racemosus* and found that a concentration of 0.8 *μ*g/mL was effective in the complete inhibition of morphogenesis in *M. racemosus* [37]. Our findings supported the hypothesis that enhancement of *β*-carotene accumulation was inversely correlated with total lipid percentage in CBS 277.49 by redirecting metabolic flux of acetyl-CoA in CBS 277.49. This was according to Miao et al. who also reported an inverse correlation between FAS and the mevalonate pathway [20].

### 3.4. Effect of Ketoconazole on Growth, Ergosterol, and *β*-Carotene Production

It has been described in the literature that carotenoid and ergosterol are part of the highly conserved mevalonate pathway in *M. circinelloides* [41]. These two-branched pathways share FPP, which is a common precursor of sterol and *β*-carotene synthesis. Hence, we speculated that enhanced production of *β*-carotene could be achieved by redistribution of FPP from sterol towards the carotenoid biosynthesis pathway by using ketoconazole in CBS 277.49. To test this hypothesis, ketoconazole was used in different concentrations based on the previous literature [42]. Since the initial addition of ketoconazole can significantly affect the growth, so it was added in media after the late exponential phase of cell growth. Ketoconazole is a potent antifungal agent that works by inhibiting the enzyme cytochrome P-450-dependent C14 demethylase that carries out the conversion of lanosterol to ergosterol [43]. *Mucor* spp. were reported to produce 10.82 *μ*g/mg of ergosterol [44].

**Figure 5:**
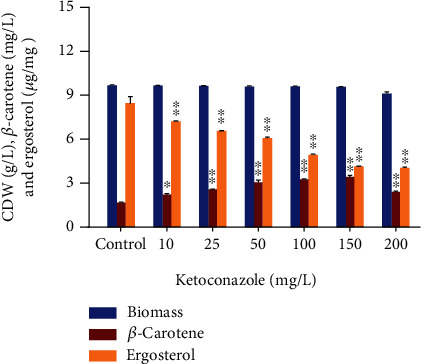
Effect of different concentrations of ketoconazole on the growth, *β*-carotene, and ergosterol production of CBS 277.49. Values are the mean ± standard deviation of three experiments. Asterisks indicate that the difference is significant in comparison to control (0) (^∗^*p* < 0.001; ^∗∗^*p* < 0.0001).

In our study, ergosterol quantitation by HPLC found that with the increasing concentration of ketoconazole from 10 to 200 mg/L, ergosterol concentration was reduced from 8.45 *μ*g/mg in CBS 277.49, as shown in Figure 5. The increasing absorption of ketoconazole was found to stimulate the production of *β*-carotene in CBS 277.49 by diverting FPP towards carotenoid synthesis. The optimal production of *β*-carotene corresponding to 3.45 mg/L (0.35 mg/g) was achieved at 150 mg/L ketoconazole, accompanied by a reduction of ergosterol concentration (4.16 *μ*g/mg) without having negative effect on growth. This value corresponded to a 105% increment in *β*-carotene production in comparison to the control culture (1.68 mg/L) in which no ketoconazole was added. It was observed that further increasing the concentration (200 mg/L) has slightly affected the growth and *β*-carotene production of *Mucor* (Figure 5).

Our results recommended that enhancement of *β*-carotene in CBS 277.49 was due to the arrest of ergosterol biosynthesis by ketoconazole and refluxing more FPP intermediate towards the branched carotenoid biosynthesis pathway. Similar to our findings, previous studies also successfully elucidated the 15% enhancement of *β*-carotene accumulation by using 100 mg/L of ketoconazole in the recombinant strain of *S. cerevisiae* [42]. Miao et al. blocked the ergosterol synthesis by 60 mg/L of fluconazole (ergosterol inhibitor) with five-fold increment in astaxanthin production in wild *P. rhodozyma*, concluding the inverse relationship of astaxanthin synthesis with fatty acid and ergosterol [20]. In another study, a genetic approach was used to downregulate the squalene pathway in *S. cerevisiae*, thereby redirecting more FPP to stimulate the synthesis of terpenoid of interest [45]. Based on these pieces of evidence, we concluded that ketoconazole effectively arrested the biosynthesis of ergosterol, resulting in increased production of carotenoid without affecting the growth of CBS 277.49.

The successful utilization of cerulenin and ketoconazole was indicated by the reduction of total lipid and ergosterol, respectively, which boosted the production of *β*-carotene in CBS 277.49 strain. This might be ascribed to the accumulation of more acetyl-CoA and FPP precursors in mevalonate metabolic flux. Our screening experiments found that modulation of the C/N ratio, cerulenin, and ketoconazole successfully augmented the *β*-carotene production to some extent, which might be due to the shift of the metabolic flux from FAS and sterol biosynthesis toward *β*-carotene production. Nevertheless, it could be interesting to investigate whether the synergistic effect of these factors could potentially amplify the production, thus further alleviating the *β*-carotene production by CBS 277.49 using RSM.

### 3.5. Synergistic Effect of the C/N Ratio, Cerulenin, and Ketoconazole for Enhanced *β*-Carotene Production in CBS 277.49

In the present study, the synergistic effect of the C/N ratio, cerulenin, and ketoconazole was checked and optimized for superior *β*-carotene production by CBS 277.49, using RSM-CCD. RSM is a powerful statistical tool that has been used successfully in many biological and chemical processes for optimization [46]. It creates an experimental design with a minimal number of experiments. It generates a model with the capability to predict the interaction between a set of independent variables and observed effects. It can generate tailored condition that differs from the standard optimization approach, which explores only one factor at a time and is considered laborious and time-consuming [47]. The concentration range of three factors was selected on the basis of results obtained from the screening experiment, as shown in Figures 3, 4, and 5. Twenty sets of experiments were conducted by using the different combinations of factors, and the mean result of three independent replicates for *β*-carotene production (experimental and predicted) is shown in Table 2.

**Figure 6:**
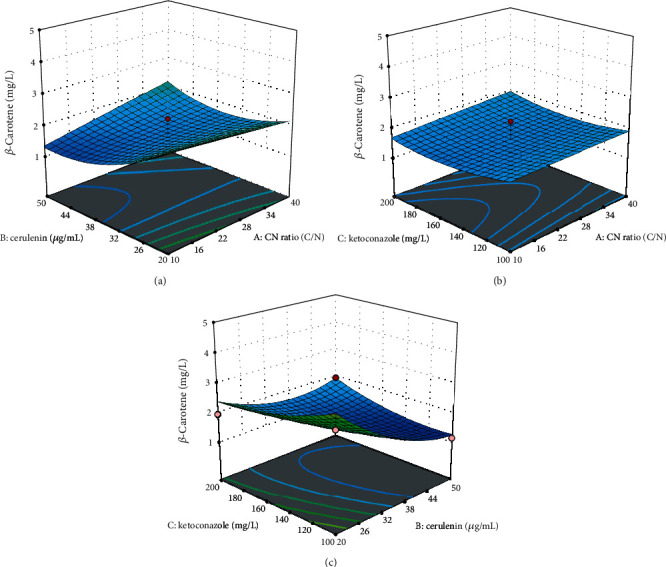
Response surface plots of *β*-carotene produced by the interaction of (a) C/N ratio and cerulenin concentration, (b) C/N ratio and ketoconazole, and (c) cerulenin and ketoconazole for *β*-carotene production by CBS 277.49.

The results showed that the synergistic effect of C/N ratios, cerulenin, and ketoconazole at different concentrations significantly affected the *β*-carotene productions by CBS 277.49 (Table 2). The experimental results for *β*-carotene production were also comparable to those of the predicted values, indicating the suitability of the design for optimization. Maximum *β*-carotene production of 4.26 mg/L (0.43 mg/g) was achieved at std. order 11 with the combination of C/N 25, 10 *μ*g/mL cerulenin, and 150 mg/L ketoconazole. On the other hand, other combinations of factors were shown to be lower than those of individual supplementation of the C/N ratio, cerulenin, and ketoconazole which achieved maximum production of 3.29 (0.31 mg/g), 3.23 (0.31 mg/g), and 3.45 mg/L (0.35 mg/g) at C/N 20, 40 *μ*g/mL, and 150 mg/L, respectively. Similarly, the interaction of factors that were visualized by the three-dimensional (3D) response surface also recorded the same pattern where minimal *β*-carotene production range between 1.5 and nearly 3.0 mg/L was observed (Figure 6). Thus, further optimization was conducted to evaluate the optimal concentration of the three factors proposed by the RSM software.

Five models were tried, such as mean, linear, 2FI, cubic, and quadratic, of which the quadratic model was selected for further analysis as it had the highest-order polynomial with a significant sum of squares, the highest *R*^2^, and an insignificant lack of fit (Supplementary Table 1). Based on the ANOVA analysis, the experimental values gained from the design were regressed using a quadratic polynomial equation, and the regression equation, expressed in terms of the coded variables, is described below:

(3) $$\begin{array}{c} \beta ‐\text{carotene}\left(\text{mg}/\text{L}\right)=+1645.06+107.53\times A–278.82\times B–14.09\times C–3.12\times A^{2}+420.05\times B^{2}+227.87\times C^{2}+315.94\times A\times B+33.30\times A\times C+391.41\times B\times C. \end{array}$$

The optimum levels of the variables for *β*-carotene production were calculated based on the regression analysis of the model. The condition proposed by the quadratic model was C/N 22, 20 *μ*g/mL cerulenin, and 118 mg/L ketoconazole with the predicted *β*-carotene production of 2.90 mg/L (0.28 mg/g).

### 3.6. Model Validation

**Figure 7:**
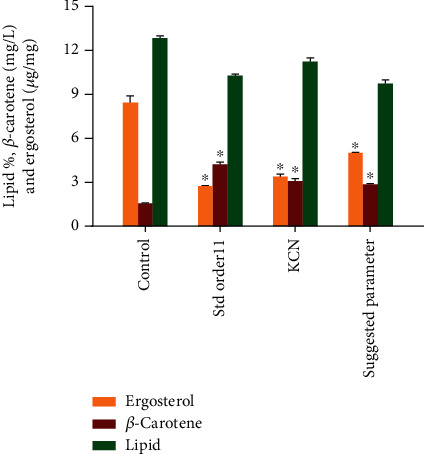
Comparative description of suggested parameters, std. order 11, KCN, and control (standard K&R medium) for ergosterol, *β*-carotene, and lipid production in CBS 277.49. Asterisks indicate that the difference is significant in comparison to control (^∗^*p* < 0.0001).

To validate the predicted value of *β*-carotene production by CBS 277.49, further experiments were carried out, under the estimated suggested conditions created by the software and compared to the std. order 11 (maximum production of *β*-carotene), KCN (similar composition to the std. order 11, but the cerulenin was omitted), and control (standard K&R medium) (Figure 7). The result showed that the *β*-carotene values obtained from RSM-suggested conditions 2.97 mg/L (0.28 mg/g) were comparable to the predicted values proposed by the model. Hence, this experiment validated the model, and CCD can be used as an optimization approach to enhance the production of *β*-carotene in CBS 277.49.

Nevertheless, although the *β*-carotene production under the suggested condition was approximately 76.80% higher as compared to the control, it was significantly lower than that of the std. order 11, which achieved nearly 4.26 mg/L (0.43 mg/g) of *β*-carotene production (Figure 7). The model is, however, unable to generate a similar condition, as shown in std. order 11 as the cerulenin concentration falls within the –*α*, outside the subjected ranges of the factor, which was between 20 and 50 *μ*g/mL (Table 1). Since the C/N and ketoconazole concentration in std. order 11 and the optimal condition proposed by the software are comparable, the augmented *β*-carotene production observed in std. order 11 might be attributed to the lower concentration of cerulenin (10 *μ*g/mL). This observation was different than the individual supplementation of cerulenin, where moderate concentration (40 *μ*g/mL) of cerulenin was needed to obtain maximum *β*-carotene production. However, when the cerulenin was omitted from the mixture (KCN), the *β*-carotene production dropped noticeably as compared to the std. order 11, signifying the role of cerulenin in the mixture to achieve maximum *β*-carotene production by CBS 277.49 (Figure 7).

The maximum *β*-carotene production achieved by individual, synergistic, and optimal factors proposed by the model is summarized in Table 3. The result showed that individual factors have more significant impact on *β*-carotene production compared to the RSM-suggested parameters and KCN. Nevertheless, a synergistic combination of factors in std. order 11 increased the *β*-carotene production by 157% compared to the control and was significantly higher than the individual supplementation, proposing that we have successfully redirected the metabolic flux towards the mevalonate pathway for higher *β*-carotene production in *M. circinelloides* CBS 277.49.

In the recent few years, the production of *β*-carotene has increased worldwide due to vast applications in various industries. Currently, remarkable achievements have been made in the optimization of medium components, and the optimal composition of media proved to be a key factor for the commercialization of the fermentation operation [32]. Even though it is the first time that the optimization of carotenoids using a metabolic flux redirecting strategy has been conducted in *M. circinelloides*, several previous studies demonstrated the metabolic flux redistribution in other microorganisms [20, 42]. Bhosale and Bernstein reported the maximum *β*-carotene level corresponding to 7.85 mg/L by *Flavobacterium multivorum* with an optimized medium [48]. Malisorn and Suntornsuk were able to produce only 201 *μ*g/L of *β*-carotene by *R. glutinis* using face-centered composite design of RSM by using radish bran as a carbon source [49]. Choudhari et al. successfully used RSM to describe the optimal value of selected variables for higher production by *B. trispora* and obtained a substantial enhancement in *β*-carotene production that corresponded to 139 mg/L [50]. In another research, only 30% augmentation in carotene production was observed as compared to the unoptimized medium in *R. glutinis* DM28 using CCD [51]. Recently, Abdelhafez et al. also carried out an optimization of *β*-carotene production using the Plackett–Burman design and CCD-RSM by *Serratia marcescens* ATCC 27117 and achieved 2.24 mg/L of *β*-carotene in their study [29]. It has been documented in another study that genetically engineered *Y. lipolytica* could produce 4 g/L of *β*-carotene using fed-batch fermentation with optimized media [18].

Thakur and Azmi reported 3.4 mg/L of *β*-carotene production in *Mucor azygosporus* MTCC 414 by cultivating it on malt yeast peptone medium [28]. In another investigation, *β*-carotene production of nearly 14 *μ*g/g by *M. circinelloides* was achieved [41]. Zhang et al. reported that the nonmodified strain of *M. circinelloides* R7B produced 170.8 *μ*g/g of *β*-carotene [11]. Similarly, in another investigation, the wild strain of *M. circinelloides* 277.49 produced 257 *μ*g/g of *β*-carotene [52]. *β*-Carotene production by different species of *Mucor* is shown in Table 4.

## 4. Conclusion

The present study optimized the medium components for attaining high *β*-carotene accumulation by *M. circinelloides* CBS 277.49 in shake flask fermentation. RSM was successfully employed to get a higher possible increment of *β*-carotene in this fungal strain by using a push and pull metabolic strategy with the help of FAS and sterol biosynthesis pathways inhibitor. Under the synergistic combinations of the studied factors, 4.26 mg/L (0.43 mg/g)of *β*-carotene was produced as compared to control, which only showed 1.68 mg/L (0.17 mg/g)of *β*-carotene production. The result suggested that RSM has the benefit of recognizing the most significant medium components and their optimal levels and was useful for functioning the fermentation process towards the accumulation of the desired metabolite. Furthermore, it was also suggested that being a model strain of carotenogenesis and valuable lipid production, *M. circinelloides* can be used as a promising zygomycete after medium optimization and genetic improvement for the biotechnological production of *β*-carotene and lipid.

## Figures and Tables

**Table 1:** Coded values of independent variables.

Independent variables	Code levels				
	-*α*	-1	0	+1	+*α*
A: CN ratios (*w*/*w*)	0.00	10.00	60.00	40.00	50.00
B: cerulenin (*μ*g/mL)	10.00	20.00	35.00	50.00	60.35
C: ketoconazole (mg/L)	66.00	100.00	150.00	200.00	235.00

**Table 2:** Design matrix of medium optimization for *β*-carotene production in CBS 277.49.

Std. order	Variables			Results	
	C/N ratios	Cerulenin (*μ*g/mL)	Ketoconazole (mg/L)	Observed values	Predicted values
				*β*-Carotene (mg/L)	*β*-Carotene (mg/L)
1	10	20	100	2.93	3.21
2	40	20	100	2.24	2.73
3	10	50	100	1.09	1.24
4	40	50	100	1.91	2.02
5	10	20	200	1.96	2.33
6	40	20	200	1.65	1.98
7	10	50	200	1.93	1.93
8	40	50	200	2.84	2.84
9	0	35	150	1.81	1.45
10	50	35	150	2.14	1.81
11	25	10	150	4.26	3.80
12	25	60.3	150	2.27	2.36
13	25	35	66	2.80	2.31
14	25	35	235	2.45	2.26
15	25	35	150	2.24	1.64
16	25	35	150	1.49	1.64
17	25	35	150	1.51	1.64
18	25	35	150	1.49	1.64
19	25	35	150	1.49	1.64
20	25	35	150	1.50	1.64

**Table 3:** Comparison of *β*-carotene production and percentage increment, with the control set as the reference of the individual and synergistic combination of factors. Values represent the means of triplicate experiments (*n* = 3).

Treatment	Concentration	*β*-Carotene, mg/L (mg/g)	% increment
Control (K&R media)	80 g/L glucose	1.68 (0.17 mg/g)	—
C/N	20	3.29 (0.31 mg/g)	95.83
Cerulenin	40 *μ*g/mL	3.23 (0.31 mg/g)	92.3
Ketoconazole	150 mg/L	3.45 (0.35 mg/g)	105.36
RSM-suggested combination	C/N 22+cerulenin 20 *μ*g/mL+ketoconazole 118 mg/L	2.97 (0.28 mg/g)	76.79
KCN	C/N 25+cerulenin 0 *μ*g/mL+ketoconazole 150 mg/L	3.20 (0.32 mg/g)	90.48
Std. order 11	C/N 25+cerulenin 10 *μ*g/mL+ketoconazole 150 mg/L	4.26 (0.43 mg/g)	157.15

**Table 4:** *β*-Carotene production by different *Mucor* spp.

Microorganisms	*β*-Carotene	References
*M. hiemalis*	1.33 mg/g	[53]
*M. circinelloides*	0.69 mg/g	[21]
*M. circinelloides* M20	0.37 mg/g	[9]
*M. rouxii*	0.19 mg/g	[9]
*M. circinelloides R7B*	0.17 mg/g	[11]
*M. circinelloides* WJ11	0.27 mg/g	[21]
*M. circinelloides* AUMC 698	0.25 mg/g	[54]
*M. circinelloides* CBS 277.49	0.43 mg/g	This study

## Data Availability

No data were used to support this study.

## Supplementary Materials

Supplementary Materials Supplementary Table 1: ANOVA for the quadratic model.
