# Supplementary material for: Redirecting Metabolic Flux towards the Mevalonate Pathway for Enhanced β-Carotene Production in M. circinelloides CBS 277.49
Source: Biomed Res Int. 2020 Dec 29;2020:8890269. doi: 10.1155/2020/8890269 (PMC7785371; doi:10.1155/2020/8890269)
Supplement: Supplementary Materials — Supplementary Table 1: ANOVA for the quadratic model. [file 8890269.f1.docx]

**Supplementary Table 1**: ANOVA analysis for the quadratic model

| **Sources** | **Sum of Squares** | **DF** | **Mean Square** | **F Value** | **Prob > F** |
| --- | --- | --- | --- | --- | --- |
| Model | 6.356E+006 | 9 | 7.062E+005 | 3.03 | 0.0495 |
| A | 1.573E+005 | 1 | 1.573E+005 | 0.67 | 0.4305 |
| B | 1.062E+006 | 1 | 1.062E+006 | 4.56 | 0.0586 |
| C | 2712.27 | 1 | 2712.27 | 0.012 | 0.9162 |
| A^2^ | 138.44 | 1 | 138.44 | 5.940E-004 | 0.9810 |
| B^2^ | 2.544E+006 | 1 | 2.544E+006 | 10.91 | 0.0080 |
| C^2^ | 7.486E+005 | 1 | 7.486E+005 | 3.21 | 0.1034 |
| AB | 7.986E+005 | 1 | 7.986E+005 | 3.43 | 0.0939 |
| AC | 8869.41 | 1 | 8869.41 | 0.038 | 0.8492 |
| BC | 1.226E+006 | 1 | 1.226E+006 | 5.26 | 0.0448 |
| Residual | 2.331E+006 | 10 | 2.331E+005 |  |  |
| Lack of Fit | 1.864E+006 | 5 | 3.728E+005 | 4.00 | 0.0774 |
| Pure Error | 4.666E+005 | 5 | 93316.61 |  |  |
| Cor Total | 8.687E+006 | 19 |  |  |  |
| Std. Dev. | 482.71 |  | R^2^ | 0.73 |  |
| Adeq. Precision | 6.03 |  | Adj R^2^ | 0.50 |  |
